# Supplementary material for: Influence of Cortisol on the Fibril Formation Kinetics of Aβ42 Peptide: A Multi-Technical Approach
Source: Int J Mol Sci. 2022 May 26;23(11):6007. doi: 10.3390/ijms23116007 (PMC9180743; doi:10.3390/ijms23116007)
Supplement: Supplementary file 1 [file ijms-23-06007-s001.zip › ijms-1622713-supplementary.pdf]

International Journal of *Molecular Sciences*

MDPI

Supplementary Materials

Influence of cortisol on the fibril formation kinetics of Aβ42 peptide: a multi-technical approach

Alessandro Nucara[1], Francesca Ripanti[2*], Simona Sennato[3], Giacomo Nisini[1], Emiliano De Santis[4], Mahta Sefat[5], Marina Carbonaro[6], Dalila Mango[5,7], Velia Minicozzi[8], Marilena Carbone[9]

1 Department of Physics, Sapienza University of Rome, P.le A. Moro 5, 00185 Rome, Italy
2 Department of Physics and Geology, University of Perugia, Via Alessandro Pascoli, 06123 Perugia, Italy
3 CNR-Institute of Complex Systems (ISC)-Sede Sapienza c/o Physics Department, Sapienza University, P.le A. Moro 5, Rome, Italy
4 Department of Physics and Astronomy and Department of Chemistry, BMC – Uppsala & University - Husargatan 3, 752 37 Uppsala, Sweden
5 School of Pharmacy, Tor Vergata University of Rome, Via della Ricerca Scientifica 1, 00133 Rome, Italy
6 Council for Agricultural Research and Economics (CREA), Research Centre for Food and Nutrition, Via Ardeatina 546, 00178 Rome, Italy
7 Laboratory Pharmacology of Synaptic Plasticity, European Brain Research Institute, 00161 Rome, Italy
8 Department of Physics, Tor Vergata University of Rome, Via della Ricerca Scientifica 1, 00133 Rome, Italy
9 Department of Chemical Science and Technologies, Tor Vergata University of Rome, Via della Ricerca Scientifica 1, 00133 Rome, Italy

* corresponding authors: francesca.ripanti@unipg.it, alessandro.nucara@uniroma1.it

S1. Fit to IR data: parameters and residues

		β-parallel/ antiparallel	α-helix	random coil	turn	β-antiparallel
A	Sec. structure fraction**	0.056 ± 0.004	0.88 ± 0.01	---	0.039 ± 0.005	0.024 ± 0.005
	Central frequency	1625 ± 2	1657 ± 8	---	1672 ± 2	1698 ± 2
	Width	8.8 ± 0.4	22 ± 2	---	8 ± 1	9.5 ± 2
B	Sec. structure fraction	0.02 ± 0.01	0.48 ± 0.05 ***	0.49 ± 0.05	---	< 0.01
	Central frequency	1627 ± 2	1669 ± 9	1648 ± 4	---	1694 ± 2
	Width	6.0 ± 0.5	17± 2	17± 3	---	14 ± 6
C	Sec. structure fraction	0.49 ± 0.09	---	0.17 ± 0.05	---	0.34 ± 0.05
	Central frequency	1627 ± 2	---	1648 ± 2	---	1686 ± 2
	Width	10± 2	---	7± 2	---	10± 2
D	Sec. structure fraction	0.16 ± 0.02	0.58 ± 0.05	---	0.18 ± 0.02	0.08 ± 0.02
	Central frequency	1626 ± 2	1654 ± 2	---	1666 ± 8	1693 ± 2
	Width	10 ± 2	8 ± 5	---	15 ± 5	10 ± 2
E	Sec. structure fraction	0.09 ± 0.02	< 0.01	0.89 ± 0.02	---	< 0.01

	Central frequency	1629 ± 2	1653± 5	1649 ± 5	---	1694 ± 2
	Width	10 ± 2	8 ± 3	32 ± 5	---	7 ± 2
F	Sec. structure fraction	0.07 ± 0.02	---	0.89 ± 0.05	0.03 ± 0.02	< 0.01
	Central frequency	1628 ± 2	---	1649 ± 5	1667 ± 5	1689 ± 2
	Width	8 ± 2	---	9 ± 2	30 ± 2	8 ± 2

Table S1. Gaussian parameters of the fits reported in Figure 2 of the manuscript. Frequencies and widths are in cm^{-1}. ** this parameter is obtained accounting for a scaling as suggested in Ref. [S1].
*** indistinguishable from the turn bands.

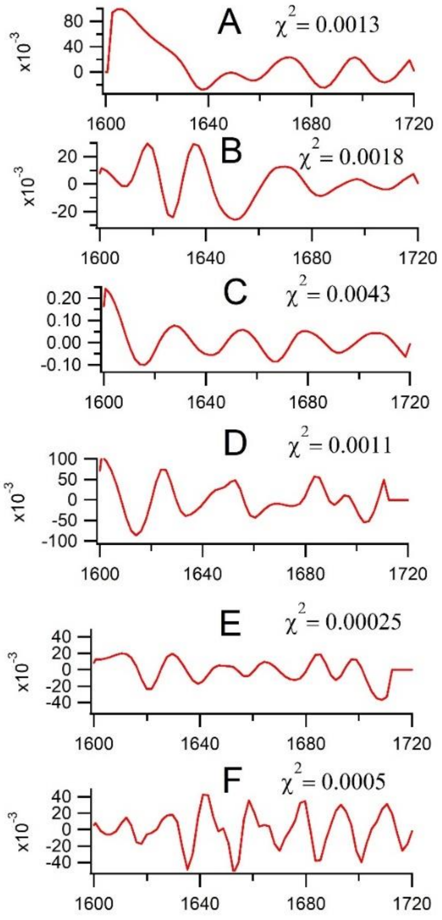

Figure S1. Residues and χ^2 values of the fit to IR data reported in Figure 2, from sample A to F.

S2. Molecular Dynamics results

The percentage of secondary structure components for Aβ42 monomers in the absence and in the presence of cortisol molecules was calculated for each sample and its time evolution was computed for 200 ps. As secondary structure components, we considered only random coil, helices, and β-structures. The results are reported in Figure S2.

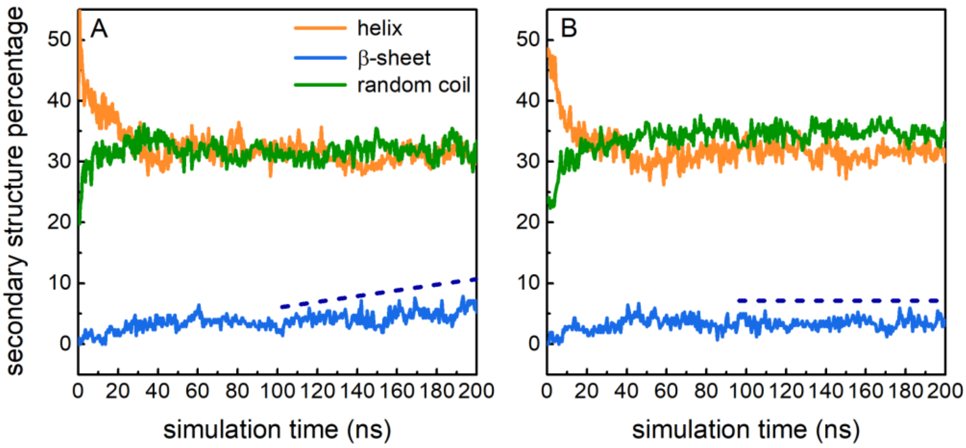

Figure S2. Secondary structure component percentage as a function of simulation time in the absence (A) and in the presence (B) of cortisol. Dotted lines mark the β-sheet percentage fit in the last 100 ns of simulation.

The contact matrices shown for the time frames involving the first and the last 50 ns of simulation are reported in Figure S3. In the presence of cortisol, the intramolecular contacts among the Aβ42 monomers are formed since the very early stages of the simulations (upper diagonal matrix in the left panel). At the end of the simulations formation of almost the same number of contacts independently of the presence of cortisol molecules are present, but cortisol-Aβ42 complexes form larger aggregates. The time evolution of contacts of cortisol with Aβ42 peptides are shown in Figure S4.

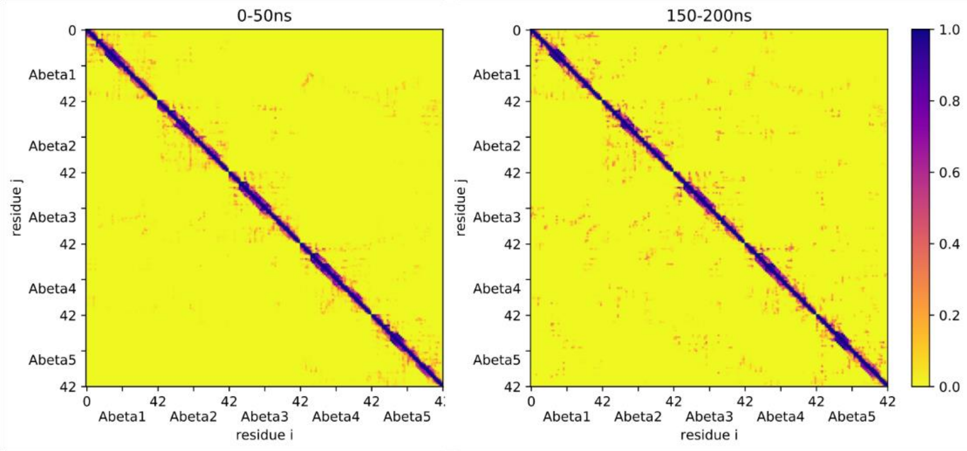

Figure S3. Average normalized matrices of contacts. A contact is defined if at least one atom of Aβ42 residue is at a distance equal or lower to 3.5 from any atom of any cortisol molecules. The average is performed in the first 50 ns (left panel) and in the last 50 ns (right panel) of simulations times and over all the three replicas. The normalization is performed in order to have a value of 1 for a contact that is present in all the three replicas and in all the frames of the analyzed portion of the simulation. Lower triangular matrices represent the contacts in the simulations where only Aβ42 peptides are present. Upper triangular matrices show the contacts for the simulations of the systems comprising Aβ42 peptides and cortisol molecules.

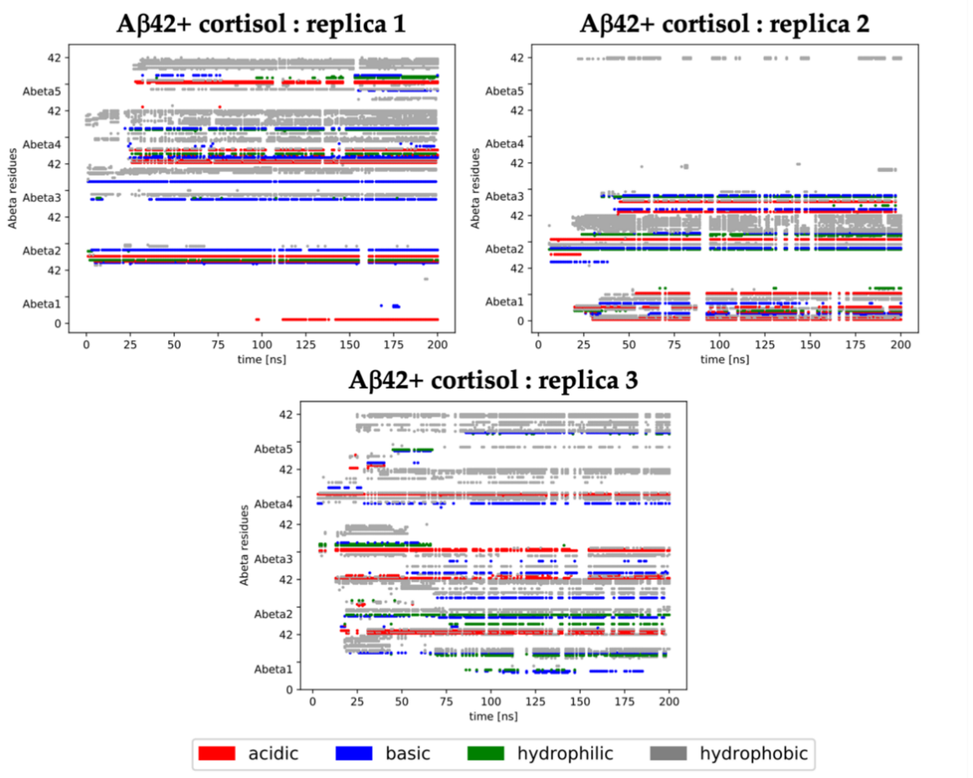

Figure S4. Time evolution of the contacts among cortisol molecules and Aβ42 residues for each of the simulated replicas. A contact is defined as in Figure S3. Acidic residues are colored in red, basic residues are in blue, hydrophilic residues are in green, and hydrophobic residues are in gray.

**Aβ42 primary structure**<br>DAEFRHDSGYEVHHQKLVFFAEDVGSNKGAIIGLMVGGVVIA		
Amino acid type	Percentage of contacts with cortisol	Percentage of amino acid type
Acidic (D, E)	16%	14%
Basic (H, K, R)	14%	14%
Hydrophilic (N, Q, S)	9%	10%
Hydrophobic (A, F, G, I, L, M, V, Y)	61%	62%

Table S2 In the first row Aβ42 peptide sequence is reported. Percentage of contacts among Aβ42 residues and cortisol molecules are classified in four groups according to their chemical properties and compared with the percentage of the amino acid type content. Amino acids belonging to each group are listed in brackets. Residues groups are colored as in Figure S4.

S3. Fluorescence data and fitting curves

Data and fits for ρ = 0.2 and ρ = 0.33 are reported in Figure S5. **According to statistical criteria, the two data sets can be satisfactory reproduced by an identical Boltzmann function.**

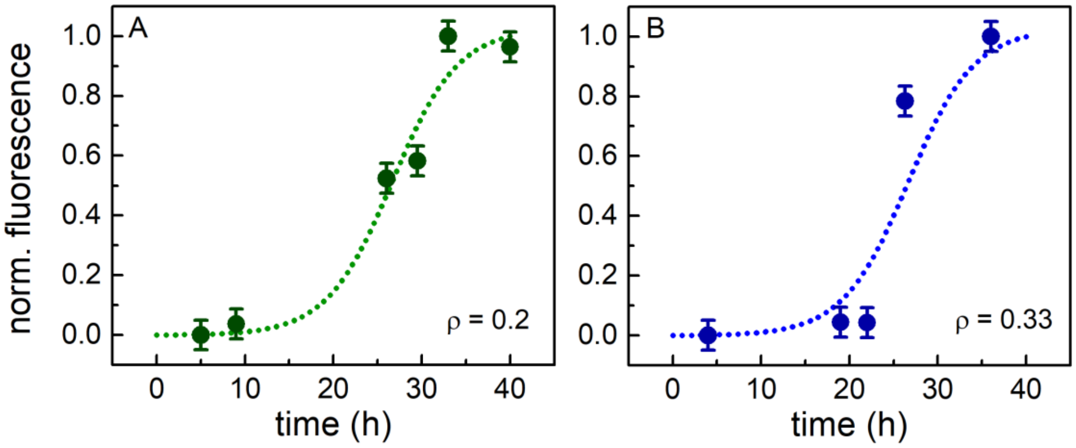

Figure S5. Normalized ThT fluorescence intensity as function of time with **the same** Boltzmann fit for ρ = 0.2 (A) and ρ = 0.33 (B).

Moreover, in order to evaluate the reproducibility of our experiments, we report in Figure S6 the normalized fluorescence intensity of two different data sets acquired on Aβ42 peptide solution.

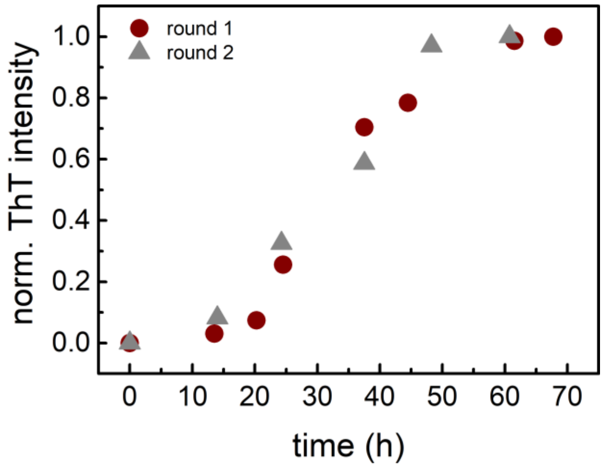

Figure S6. Normalized ThT fluorescence intensity as function of time for two different data sets acquired on the Aβ42 peptide (c = 5 μM in 4PBS:1DMSO buffer).

S4. Statistical analysis of AFM images

AFM height distribution functions of the images shown in Figure 6 (main text), for native Aβ42 sample after 40 h (A) and 78 h (B, C) of incubation in 4PBS:1DMSO, are reported in Figure S7. The distribution functions are computed by Gwiddion software as normalized histograms of all the height values of each AFM image.

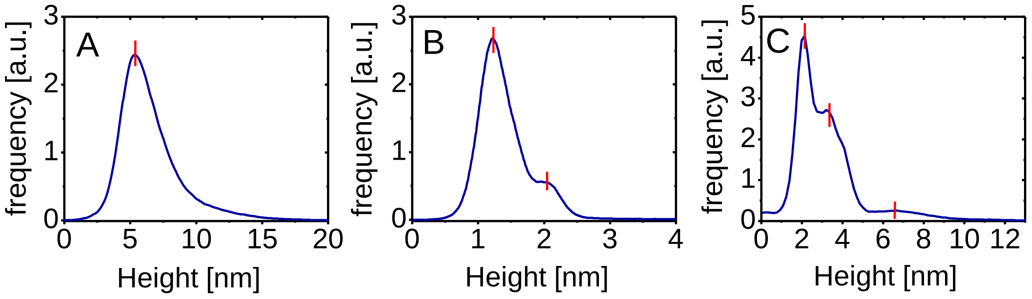

Figure S7. AFM height distribution functions for native Aβ42 sample after 40 h (A) and 78 h (B, C) of incubation in 4PBS:1DMSO, calculated on the images shown in Figure 6 of the manuscript. Red lines identify the peaks of the distributions.

Reproducibility of AFM results was evaluated by performing a statistical analysis on three different images, at least, obtained by independent deposition of each sample. The height histograms were calculated by considering the height profile of 100-150 different structures, individually selected on the different images collected for each sample. In the case of Aβ42 sample incubated at 78 h, fibrils and worm-like objects were counted separately, as they are representative of the different structures found in this condition. Results of this analysis are shown in Figure S8.

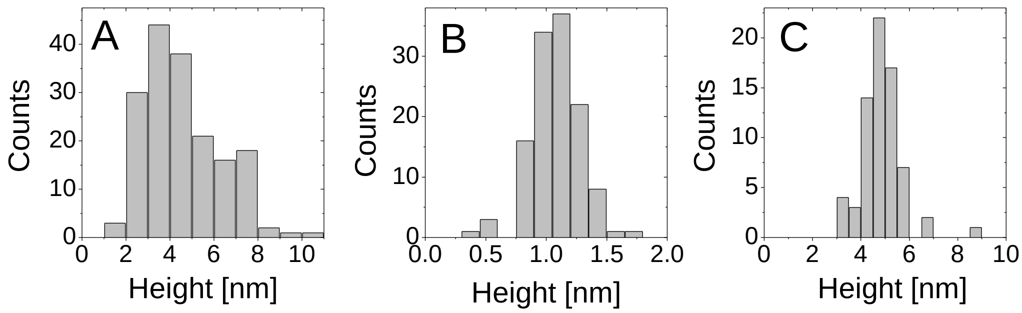

Figure S8. Height histograms for native Aβ42 sample after 40 h (A) and 78 h (B, C) of incubation in 4PBS:1DMSO, calculated on typical structures of each sample, on three different images, at least.

The mean values of the histograms reported in Figure S8 and values of the height distribution peaks reported in Figure S7 are shown in Table S3. The mean values of the height of the typical structures identified by direct selection in each sample are in agreement with those obtained by calculating the height distribution on the whole image. This result gives evidence of the reproducibility of the analysis and eliminates any problems connected to the biased choice of structures to be counted or AFM data manipulation. As it has to be expected, the height distribution calculated on the whole AFM image is able to capture the presence of all the structures present in each image by distinguishing one or more peaks, if present. This is indeed what we observed for Aβ42 incubated 78 h, where the heterogeneity of the sample is reflected by the coexistence of the structures with distinct height distribution.

	**Aβ42 – 40 h**	**Aβ42 – 78 h**	**Aβ42 – 78 h**
mean ± sd_{mean} - histogram<br>[collection of images]	4.53 ± 0.13	1.079 ± 0.019	4.834 ± 0.010
peak – height distribution<br>[single image]	5.21 ± 0.13	1.260 ± 0.032<br>2.15 ±.010	2.09 ± 0.10<br>3.21 ± 0.16<br>6.65 ± 0.25

Table S3. Statistical Analysis of AFM data: values of the peak maximum of the height distributions shown in Figure 6 (main text) and mean value of the histograms calculated for typical structures characterizing the Aβ42 sample after 40 h and 78 h incubation time. The values of the peaks of the height distribution were found by multi-peak analysis by Gwiddion software.

The statistical analysis was carried out also for the Aβ42 samples in the presence of cortisol, by considering the images shown in Figure 7 of the manuscript. The results of this analysis are here reported in Figures S9, S10, and in Table S4.

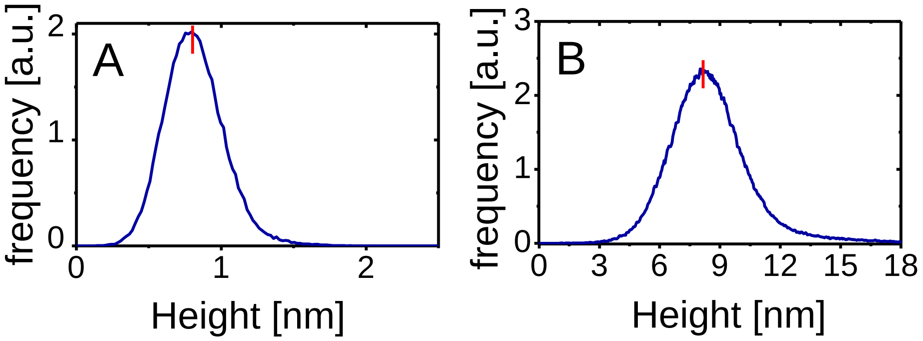

Figure S9. AFM height distribution functions for Aβ42 samples at ρ = 0.1 (A) and ρ = 1 (B), calculated on the images shown in Figure 7 of the manuscript. Red lines identify the peaks of the distributions.

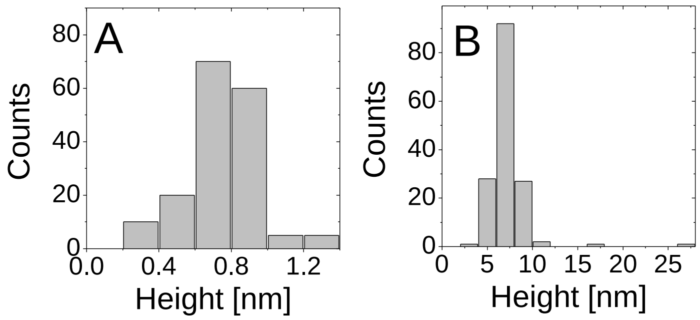

Figure S10. Height histograms for Aβ42 samples at ρ = 0.1 (A) and ρ = 1 (B).

	$\rho = 0.1$	$\rho = 1$
mean ± sd_{mean} - histogram [collection of images]	0.721 ± 0.036	7.18 ± 0.17
peak - height distribution [single image]	0.790 ± 0.013	8.170 ± 0.092

Table S4. Statistical analysis of AFM data: values of the peak maximum of the height distributions shown in Figure 7 (main text) and mean value of the histograms calculated for typical structures characterizing the Aβ42 sample in the presence of cortisol at $\rho = 0.1$ and $\rho = 1$. The values of the peaks of the height distribution were found by multi-peak analysis by Gwiddion software.

References

S1. Ripanti, F.; Luchetti, N.; Nucara, A.; Minicozzi, V.; Di Venere, A.; Filabozzi, A.; Carbonaro, M. Normal mode calculation and infrared spectroscopy of proteins in water solution: Relationship between amide I transition dipole strength and secondary structure. *Int. J. Biol. Macromol.* **2021**, *185*, 369-376.
